# Parsimonious Clone Tree Integration in cancer

**DOI:** 10.1186/s13015-022-00209-9

**Published:** 2022-03-14

**Authors:** Palash Sashittal, Simone Zaccaria, Mohammed El-Kebir

**Affiliations:** 1 Department of Computer Science, University of Illinois Urbana-Champaign, Urbana, IL USA; 2 Computational Cancer Genomics Research Group, University College London Cancer Institute, London, UK; 3 Cancer Research UK Lung Cancer Centre of Excellence, University College London Cancer Institute, London, UK; 4 Cancer Center at Illinois, University of Illinois Urbana-Champaign, Urbana, IL USA

**Keywords:** Intra-tumor heterogeneity, Perfect phylogeny, Constraint programming, Single-cell DNA sequencing, Perfect phylogeny

## Abstract

**Background:**

Every tumor is composed of heterogeneous clones, each corresponding to a distinct subpopulation of cells that accumulated different types of somatic mutations, ranging from single-nucleotide variants (SNVs) to copy-number aberrations (CNAs). As the analysis of this intra-tumor heterogeneity has important clinical applications, several computational methods have been introduced to identify clones from DNA sequencing data. However, due to technological and methodological limitations, current analyses are restricted to identifying tumor clones only based on either SNVs or CNAs, preventing a comprehensive characterization of a tumor’s clonal composition.

**Results:**

To overcome these challenges, we formulate the identification of clones in terms of both SNVs and CNAs as a integration problem while accounting for uncertainty in the input SNV and CNA proportions. We thus characterize the computational complexity of this problem and we introduce PACTION (PArsimonious Clone Tree integratION), an algorithm that solves the problem using a mixed integer linear programming formulation. On simulated data, we show that tumor clones can be identified reliably, especially when further taking into account the ancestral relationships that can be inferred from the input SNVs and CNAs. On 49 tumor samples from 10 prostate cancer patients, our integration approach provides a higher resolution view of tumor evolution than previous studies.

**Conclusion:**

PACTION is an accurate and fast method that reconstructs clonal architecture of cancer tumors by integrating SNV and CNA clones inferred using existing methods.

**Supplementary Information:**

The online version contains supplementary material available at 10.1186/s13015-022-00209-9.

## Background

Cancer results from an evolutionary process where somatic mutations accumulate in the genomes of different cells. This process yields highly heterogeneous tumors composed of different *clones*, each corresponding to a distinct subpopulation of cells with the same complement of somatic mutations [1]. The resulting intra-tumor heterogeneity has been clearly linked to critically important cancer phenotypes, including cancer prognosis and the potential of developing resistance to cancer therapy [2, 3]. Therefore, important downstream applications rely on accurate reconstructions of a tumor’s clonal architecture, which in turn requires the identification of the different clones, their proportions and their evolutionary history. However, the presence of different types of somatic mutations in the same clones renders these tasks particularly challenging. In particular, the following two types of somatic mutations are frequent in cancer [4–6]: (1) single nucleotide variants (SNVs), which are substitutions of individual DNA nucleotides, and (2) copy number alterations (CNAs), which are amplifications and deletions of large genomic regions.

Most cancer sequencing studies use bulk DNA sequencing technology, where one does not directly measure the co-occurrence of different mutations in the same clone because the generated DNA sequencing reads originate from unknown mixtures of millions of different cells in a bulk tumor sample. To identify distinct clones from such data, one thus needs to deconvolve the mixed sequencing data into the different clonal components [7]. Several computational methods have been introduced to perform this task. However, the majority of existing methods only focus on either SNVs [8–12] or CNAs [13–19], but rarely on both. Methods that attempt to identify clones in terms of both SNVs and CNAs do not not scale to the numbers of current cancer sequencing datasets (e.g., number of samples, mutations, clones, etc.) and often require heuristics to reduce the size of input instances [20–22]. As a result, current cancer evolutionary analyses [23, 24] do not apply such proposed methods but rather perform a *post hoc* analysis, manually assigning CNAs to a tree inferred from SNVs. Furthermore, we note that similar issues arise with some single-cell DNA sequencing technologies, since the different features of these technologies only allow the reliable measurement of either SNVs or CNAs [25]. For example, targeted MDA single-cell sequencing technologies are more suited for the idenification of SNVs whereas whole-exome/genome DOP-PCR single-cell technologies are more suited for the identification of CNAs, and both these technologies have been used in parallel on the same tumor sample [26].

**Fig. 1:**
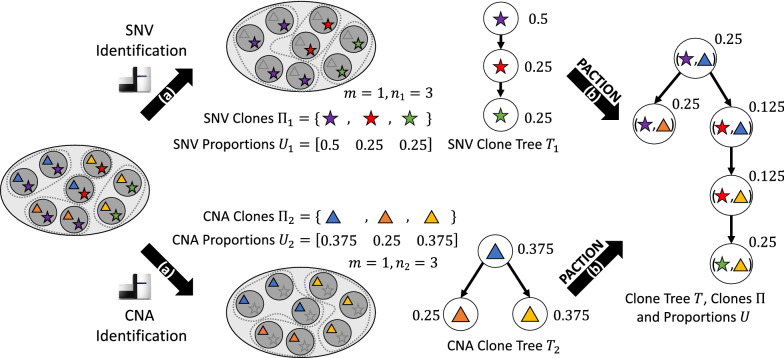
Overview. A tumor is composed of multiple subpopulations of cells, or clones, with distinct somatic mutations, which can be measured using DNA sequencing. **a** Due to limitations in inference algorithms and/or sequencing technologies, we are limited to characterizing tumor clones in terms of either single-nucleotide variants (SNVs, stars) or copy-number aberrations (CNAs, triangles). That is, we infer clones $\Pi _1$, proportions $U_1$ and a clone tree $T_1$ for the SNVs. Similarly, we infer clones $\Pi _2$, proportions $U_2$ and a clone tree $T_2$ for the CNAs. **b** PACTION solves the Parsimonious  Clone Tree Integration problem of inferring clones $\Pi \subseteq \Pi _1 \times \Pi _2$, a clone tree *T* and proportions *U* that characterize the clones of the tumor in terms of both SNVs and CNAs

In this study, we investigate whether tumor clonal compositions can be comprehensively reconstructed by an alternative simpler and automated approach. Leveraging the SNV and CNA clone proportions that can be independently and reliably inferred by existing methods, we introduce the Parsimonious Clone Integration (pci) and Parsimonious Clone Tree Integration (pcti) problems to infer clones in terms of both SNVs and CNAs, their proportions and, additionally for the pcti problem, their evolutionary relationships (Fig. 1). We prove that the proposed problems are NP-hard and we introduce PACTION (PArsimonious Clone Tree integratION), an algorithm that solves these problems using two mixed integer linear programming formulations. Using simulations, we find that our approach reliably handles errors in input SNV and CNA proportions and scales to practical instance sizes. On 49 samples from prostate cancer patients [23], we find that our approach more comprehensively reconstructs tumor clonal architectures compared to the manual approach adopted in the previous analysis of the same data.

## Problem statements

We introduce two integration problem formulations to reconstruct tumor clonal composition from inferred SNV and CNA clone proportions. The first problem aims at inferring tumor clones and related proportions with both SNVs and CNAs given the clone proportions of SNVs and CNAs independently. The second problem additionally considers phylogenetic trees describing the evolution of tumor clones with either different SNVs or CNAs.

### Parsimonious Clone Integration

Suppose a tumor is composed of a set $\Pi$ of $n = |\Pi |$ clones, which are characterised by unique complements of two different features (e.g., SNVs and CNAs). These clones occur in *m* samples at varying proportions, defined as follows.

#### Definition 1

An $m \times n$ matrix $U = [u_{p,\ell }]$ is a *proportion matrix for* *n* *clones* $\Pi$ provided (i) $u_{p,\ell } \ge 0$ for all samples $p \in [m]$ and clones $\ell \in [n]$, and (ii) $\sum _{\ell =1}^n u_{p,\ell } = 1$ for all samples $p \in [m]$.

Due to limitations in inference algorithms and/or sequencing technologies, we only infer clones and their proportions for one feature in isolation. These two features lead to two distinct partitions of all tumor cells: a set $\Pi _1 = [n_1]$ of clones induced by the first feature (e.g., SNVs) and a set $\Pi _2 = [n_2]$ of clones induced by the second feature (e.g., CNAs). We refer to the original clones as $\Pi$-clones and the clones induced by the first and the second features as $\Pi _1$-clones and $\Pi _2$-clones, respectively. The proportions of the $\Pi _1$-clones and $\Pi _2$-clones are given by the $m \times n_1$ proportion matrix $U_1 = [u^{(1)}_{p,i}]$ and the $m \times n_2$ proportions matrix $U_2 = [u^{(2)}_{p,j}]$, respectively. How are the proportions $U_1$ for $\Pi _1$-clones and the proportions $U_2$ for $\Pi _2$-clones related to the proportions *U* of the $\Pi$-clones?

**Fig. 2:**
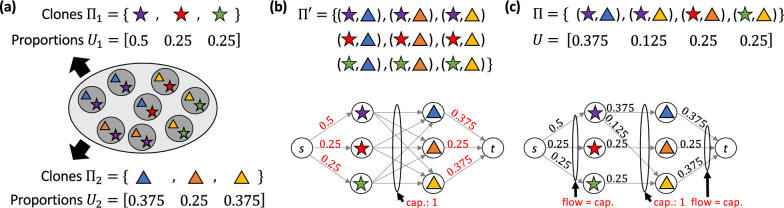
The parsimonious clone integration (pci) problem. **a** Given clones $\Pi _1$ and $\Pi _2$ and corresponding proportions $U_1$ and $U_2$, we seek clones $\Pi \subseteq \Pi _1 \times \Pi _2$ and corresponding proportions *U* consistent with $U_1$ and $U_2$. **b** There always exists a consistent proportion matrix $U'$ for the trivial solution $\Pi ' = \Pi _1 \times \Pi _2$, which can be identified by solving a maximum flow problem. **c** We seek the solution $\Pi$ with minimum number $|\Pi |$ of clones. Here, $|\Pi |=4$, which is smaller than ground truth (see panel (**a**)). The corresponding matrix *U* follows from solving the illustrated maximum flow problem. However, incorporating tree constraints, as in the pcti problem, will lead to ground truth (Fig. 1)

To answer this question, recall that $\Pi$ is a partition of all tumor cells induced by the combination of both the two features, whereas $\Pi _1$ and $\Pi _2$ are partitions induced by each feature in isolation (Fig. 2a). As such, we have that the partition $\Pi$ is a refinement of partitions $\Pi _1$ and $\Pi _2$. Thus, each $\Pi$-clone $\ell$ corresponds to a unique $\Pi _1$-clone *i* and a unique $\Pi _2$-clone *j*. In other words, we may view the set $\Pi$ as a binary relation of sets $\Pi _1$ and $\Pi _2$ of clones composed of pairs $\ell = (i,j)$ of clones, i.e., $\Pi \subseteq \Pi _1 \times \Pi _2$. This relation is captured by the projection functions $\pi _1 : \Pi \rightarrow \Pi _1$ and $\pi _2 : \Pi \rightarrow \Pi _2$ such that $\pi _1((i,j)) = i$ and $\pi _2((i,j)) = j$ for all $(i,j) \in \Pi$. We relate the proportion matrix *U* for clones $\Pi$ to the proportion matrix $U_1$ for clones $\Pi _1$ and the proportion matrix $U_2$ for clones $\Pi _2$ as follows.

#### **Definition 2**

Given projection functions $\pi _1 : \Pi \rightarrow \Pi _1$ and $\pi _2 : \Pi \rightarrow \Pi _2$ induced by the set $\Pi \subseteq \Pi _1 \times \Pi _2$ of clones, the proportion matrix $U = [u_{p,\ell }]$ for clones $\Pi$ is *consistent* with a proportion matrix $U_1 = [u^{(1)}_{p,i}]$ for clones $\Pi _1 = [n_1]$ and proportion matrix $U_2 = [u^{(2)}_{p,j}]$ for clones $\Pi _2 = [n_2]$ provided (i) $u^{(1)}_{p,i} = \sum _{\ell : \pi _1(\ell ) = i} u_{p,\ell }$ for all samples $p \in [m]$ and clones $i \in [n_1]$, and (ii) $u^{(2)}_{p,j} = \sum _{\ell : \pi _2(\ell ) = j} u_{p,\ell }$ for all samples $p \in [m]$ and clones $j \in [n_2]$.

The above definition formalizes the intuition that clones $\Pi$ of the tumor are a refinement of the input clones $\Pi _1$ and $\Pi _2$, and therefore their proportions *U* must be consistent with the input proportions $U_1$ and $U_2$. Our goal is to recover the set $\Pi \subseteq \Pi _1\times \Pi _2$ of clones and their proportions *U* from the proportion matrices $U_1$ and $U_2$ for clones $\Pi _1$ and $\Pi _2$, respectively. While there always exist trivial solutions given by the full set $\Pi ' = \Pi _1 \times \Pi _2$ of $n = n_1 \cdot n_2$ clones (Fig. 2b), we seek a solution $\Pi$ with the smallest number *n* of clones under the principle of parsimony (Fig. 2c).

#### Problem 1

(*Parsimonious Clone Integration (PCI)*) Given proportions $U_1$ for clones $\Pi _1 = [n_1]$ and proportions $U_2$ for clones $\Pi _2 = [n_2]$, find (i) the smallest set $\Pi \subseteq \Pi _1\times \Pi _2$ of clones and (ii) proportions *U* for $\Pi$ such that *U* is consistent with $U_1$ and $U_2$.

### Parsimonious Clone Tree Integration

In practice, proportions $U_1$ and $U_2$ are not measured exactly but are affected by potential measurement errors. As such, accurate recovery of the original clones $\Pi$ and their proportions *U* requires correcting $U_1$ and $U_2$. To accomplish this, we require additional information and constraints. In this work, we propose to use the evolutionary relationships among the clones $\Pi _1$ and $\Pi _2$ that can be inferred by existing methods in the form of clone trees [8, 9, 27–30]. Specifically, a rooted tree *T* is a *clone tree* for clones $\Pi$ provided the vertex set *V*(*T*) equals $\Pi$. Moreover, the root vertex *r*(*T*) of a clone tree *T* corresponds to the normal clone while each edge $(u, v) \in E(T)$ represents a mutation event that altered one of the features of clone *u* and led to the formation of the clone *v*.

Similarly to the pci problem, we are given two clone trees, one for each feature in isolation. In the specific example of two features (e.g., SNVs and CNAs), let clone tree $T_1$ describe the evolution of clones $\Pi _1$ (e.g., SNVs) and clone tree $T_2$ describe the evolution of clones $\Pi _2$ (e.g., CNAs). These trees are inferred using standard algorithms in the field [8–19]. Since all clones share a common evolutionary history, the original clone tree *T* is a *refinement* [11, 31] of the clone trees $T_1$ and $T_2$, which is defined as follows.

#### **Definition 3**

Clone tree *T* for clones $\Pi$ is a *refinement* of clone trees $T_1$ for clones $\Pi _1$ and clone tree $T_2$ for clones $\Pi _2$ provided

(i) for each edge $(i, i') \in E(T_1)$ there exists exactly one $j\in \Pi _2$ such that $((i, j), (i', j)) \in E(T)$,
(ii) for each edge $(j, j') \in E(T_2)$ there exists exactly one $i\in \Pi _1$ such that $((i, j), (i, j')) \in E(T)$,
(iii) for each $((i,j),(i',j'))\in E(T)$, it holds that $(i, i') \in E(T_1)$ and $j=j'$, or $(j, j')\in E(T_2)$ and $i = i'$.

Intuitively, the above definition states that when collapsing vertices of *T* corresponding to identical $\Pi _1$-clones one obtains $T_1$, and, similarly, $T_2$ is obtained by collapsing vertices of *T* corresponding to identical $\Pi _2$-clones.

Under a principle of parsimony and given clone trees $T_1, T_2$ with related proportions $U_1, U_2$, our goal is to find a set $\Pi \subseteq \Pi _1\times \Pi _2$ of clones, a clone proportion matrix *U*, and a $T_1,T_2$-refined clone tree *T* that require the smallest correction in $U_1$ and $U_2$. This motivates the following problem statement.

#### **Problem 2**

(*Parsimonious Clone Tree Integration (PCTI)*) Given proportions $U_1$ and tree $T_1$ for clones $\Pi _1 = [n_1]$ and proportions $U_2$ and tree $T_2$ for clones $\Pi _2 = [n_2]$, find (i) the set $\Pi$ of clones, (ii) clone tree *T* and (iii) proportions *U* for $\Pi$ such that the clone tree *T* is a refinement of $T_1$ and $T_2$ and minimizes the total error $J(U, U_1, U_2)$ such that

$$\begin{aligned} J(U, U_1, U_2) =&\sum _{p=1}^m \sum _{i=1}^{n_1} |u^{(1)}_{p,i} - \sum _{\ell :\pi _1(\ell ) = i} u_{p,\ell }| \\&+\sum _{p=1}^m \sum _{j=1}^{n_2} |u^{(2)}_{p,j} - \sum _{\ell :\pi _2(\ell ) = i} u_{p,\ell }|. \end{aligned}$$

Note that $J(U, U_1, U_2) = 0$ if and only if *U* is consistent with $U_1$ and $U_2$. The clone trees *T*, $T_1$ and $T_2$ do not appear in the objective function $J(U, U_1, U_2)$ and only provides constraints to the optimization problem. Due to these constraints, unlike the previous pci problem, pcti does not always admit a trivial solution with $J(U, U_1, U_2) = 0$ (as we further discuss in the next Section).

## Combinatorial characterization and computational complexity

We investigate the combinatorial structure and computational complexity of the two proposed pci and pcti problems in the following two sections, respectively.

### Parsimonious Clone Integration

We characterize the combinatorial structure of feasible and optimal solutions $(\Pi , U)$ for the PCI problem. We first observe that the PCI problem always has a trivial solution. Specifically, given a set $\Pi _1$ of $n_1 = |\Pi _1|$ clones and a set $\Pi _2$ of $n_2 = |\Pi _2|$ clones and corresponding proportions $U_1 \in [0,1]^{m\times n_1}$ and $U_2 \in [0,1]^{m\times n_2}$, a trivial feasible solution is composed of $n = n_1 n_2$ clones $\Pi = \Pi _1 \times \Pi _2$, which may have many possible corresponding proportions *U* (Fig. 2b). For example, proportions $U = [u_{p,(i,j)}]$ can be computed greedily by considering the *n* clones in any arbitrary order, and assigning each clone $(i,j) \in \Pi$ a proportion of $u_{p,(i,j)} = \min (u^{(1)}_{p,i}, u^{(2)}_{p,j})$ followed by subsequently updating $u^{(1)}_{p,i} := u^{(1)}_{p,i} - u_{p,(i,j)}$ and $u^{(2)}_{p,j} := u^{(2)}_{p,j} - u_{p,(i,j)}$ for each sample $p \in [m]$. Thus, $n = n_1 n_2$ is an upper bound on the number of clones needed. Can we similarly identify a lower bound on *n*?

To answer this question, let the *support* *S*(*U*) of an $m \times n$ proportion matrix *U* be defined as the number of non-zero entries in the vector $U{\mathbf {1}}_{m}$ where ${\mathbf {1}}_m$ is a $m\times 1$ vector with all entries equal to one. That is, the support *S*(*U*) of a proportion matrix *U* of clones $\Pi$ signifies the number of clones with non-zero proportion in at least one of the samples $p\in [m]$. Any such clone must be part of at least one clone $\ell \in \Pi$ in the solution to the pci problem to ensure consistency of the proportion matrices. This leads to the following observation.

#### Observation 1

Given an instance $(\Pi _1, U_1, \Pi _2, U_2)$ of the pci problem with solution $\Pi$ we have $n \ge \max (S(U_1), S(U_2))$ where $n = |\Pi |$.

Given any set $\Pi \subseteq \Pi _1 \times \Pi _2$ of clones, deciding whether there exists a proportion matrix *U* that is consistent with given proportion matrix $U_1$ for clones $\Pi _1$ and $U_2$ for clones $\Pi _2$, and constructing such a matrix is equivalent to solving a maximum flow problem, which takes polynomial time [32] (see Additional file 1: Section B). Figure 2 illustrates the construction such that there exists a consistent proportion matrix if and only the value of the flow is 1. Note that for $m>1$ samples, we need to solve a multi-commodity rather than a single-commodity flow problem. However, the pci problem, where we simultaneously seek $\Pi$ and *U*, is NP-hard and the hardness comes from having to identify the smallest set $\Pi$ of clones.

#### **Theorem 1**

*The* pci *problem is NP-hard even for number* $m=1$ *of samples*.

This follows by reduction from the 3-partition problem, a known NP-complete problem [33, 34] stated as follows.

#### **Problem 3**

(3-PARTITION) Given an integer $B\in \mathbb {N}^{>0}$, a multiset $A = \{a_1, \cdots , a_{3q}\}$ of 3*q* positive integers such that $a_i\in (B/4, B/2)$ for all $i\in [3q]$, and $\sum _{i=1}^{3q} a_i = Bq$, does there exist a partition of *A* into *q* disjoint subsets such that the sum of the integers in each subset equals *B*?

Note that since each $a_i$ occurs within the open interval (*B*/4, *B*/2) and the elements in each subset of the desired partition sum to *B*, it holds that each subset must be composed of exactly three elements from the multiset *A*—hence the name of the problem.

We represent the solution to an instance (*A*, *B*) of the 3-partition problem as a function $\sigma : [3q] \rightarrow [q]$, which encodes the division of the elements of $A = \{a_1,\ldots ,a_{3q}\}$ into *q* disjoint subsets. The inverse of this function specifies the subset corresponding to each $j\in [q]$ as $\sigma ^{-1}(j) = \{ i \in [3q] :\; \sigma (i) = j \}$. Note that any solution $\sigma : [3q] \rightarrow [q]$ of the 3-partition problem satisfies the following constraint.

1 $$\begin{aligned} \sum _{i\in \sigma ^{-1}(j)} a_{i}&= B,\quad \forall j\in [q]. \end{aligned}$$

**Fig. 3:**
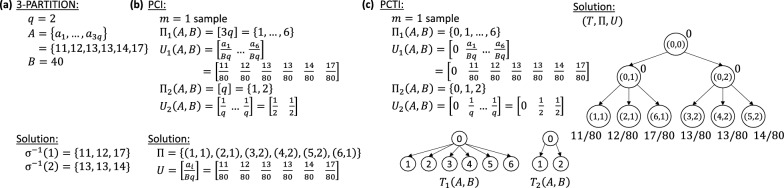
Reduction from 3-partition. **a** Example instance of 3-PARTITION with a multiset *A* of 6 elements and target sum $B=40$. **b** Corresponding pci instance $(\Pi _1,U_1,\Pi _2,U_2)$ and solution $(\Pi ,U)$. (c) Corresponding pcti instance $(T_1, \Pi _1,U_1,T_2,\Pi _2,U_2)$ and solution $(T,\Pi ,U)$

Figure 3a provides an example 3-PARTITION instance and solution.

Given a 3-partition problem instance (*A*, *B*), we construct an instance of the pci problem with number $m=1$ of samples as follows. The set $\Pi _1(A, B)$ of clones is given by the set [3*q*]. The corresponding proportions are given by the $1 \times 3q$ proportion matrix $U_1(A,B) = [u^{(1)}_{1,i}]$ where $u^{(1)}_{1,i} = a_{i}/(Bq)$ for all $i\in [3q]$. Clearly, $U_1(A, B) = [u^{(1)}_{1,i}]$ is a proportion matrix for $\Pi _1(A,B)$ as, by construction, we have that $\sum _{i=1}^{3q} u^{(1)}_{1,i} = 1$ and $u^{(1)}_{1,i} \ge 0$ for all $i\in [3q]$. The second set $\Pi _2(A, B)$ of clones is given by [*q*]. The corresponding proportions are given by the $1 \times q$ proportion matrix $U_2(A,B)=[u^{(2)}_{1,j}]$ where $u^{(2)}_{1,j} = 1/q$ for all $j\in [q]$. It is easy to verify that $U_2(A,B)$ is a proportion matrix for $\Pi _2(A,B)$. Clearly, this construction takes polynomial time. Figure 3b shows an example. Hardness follows from the following lemma whose proof is in Additional file 1: Section A.

#### **Lemma 1**

*Given proportions* $U_1(A, B)$ *for clones* $\Pi _1(A, B) = [3q]$ *and proportions* $U_2(A, B)$ *for clones* $\Pi _2(A, B) = [q]$*, there exists a set* $\Pi$ *of clones of size* $n = |\Pi | \le 3q$ *with proportions* *U* *that are consistent with* $U_1(A, B)$ *and* $U_2(A, B)$ *if and only if there exists a solution to the* 3-partition *instance* (*A*, *B*).

### Parsimonious Clone Tree Integration

We now characterize the combinatorial structure of feasible and optimal solutions $(\Pi , U, T)$ for the PCTI problem. Let $T_1$ be the first input clone tree for the input set $\Pi _1$ of $n_1 = |\Pi _1|$ clones. Similarly, let $T_2$ be the second input clone tree for the input set $\Pi _2$ of $n_2 = |\Pi _2|$ clones. Let *T* be a solution clone tree that is a refinement of both $T_1$ and $T_2$. First, we observe that the clones that label the root vertices $r(T_1)$ and $r(T_2)$ of the two input trees together label the root vertex *r*(*T*) of the output tree *T*, i.e., $r(T) = (r(T_1), r(T_2))$.

#### Observation 2

If clones $\Pi$, clone tree *T* and proportion matrix *U* form a solution to the pcti instance $(\Pi _1, T_1, U_1, \Pi _2, T_2, U_2)$, then $(r(T_1), r(T_2)) \in \Pi$ and $r(T) = (r(T_1), r(T_2))$.

Next, from Definition 3 it follows that in the output clone tree *T* it must hold that along each edge there is either a change in corresponding $\Pi _1$-clones or $\Pi _2$-clones but not both.

#### Observation 3

For each $(i,j)\in V(T) \setminus \{r(T)\}$ it holds that either $((i',j), (i,j))\in E(T)$ or $((i,j'), (i, j))\in E(T)$ where $(i', i)\in E(T_1)$ and $(j', j)\in E(T_2)$.

Combining these observations, we get that the number of vertices/clones in *T* equals $n=n_1+n_2-1$.

#### Observation 4

The number of clones *V*(*T*) equals $n = n_1 + n_2 - 1$.

We note that *T* is a multi-state perfect phylogeny with two characters, i.e. each character state labels at most one edge of *T*, whose two sets of states correspond to $\Pi _1$ and $\Pi _2$. Moreover, $T_1$ and $T_2$ impose an ordering of two sets of states to which *T* must adhere—i.e., the two characters are cladistic [35]. Additional file 1: Section C gives precise definitions of these concepts and also discusses how the problem of deciding whether there exists an error-free solution of pcti with $J(U,U_1,U_2)=0$ is equivalent to a special case of the cladistic multi-state serfect phylogeny deconvolution problem [21]. Although the tree constraints alter the solution space of pcti problem compared to the pci problem (see Figs. 1 and  2c), pcti remains NP-hard, as we will show in the following.

#### **Theorem 2**

*The* pcti *problem is NP-hard even for number* $m = 1$ *of samples.*

For a given instance (*A*, *B*) of the 3-partition problem, we construct an instance of the pcti problem as follows. The first set $\Pi _1(A, B)$ of clones equals $\{0\}\cup [3q]$ with corresponding $1\times (3q+1)$ proportion matrix $U_1(A, B) = [u^{(1)}_{1,i}]$ where $u^{(1)}_{1,i} = a_{i}/(Bq)$ for all $i\in [3q]$, and $u^{(1)}_{1,0} = 0$. The second set $\Pi _2(A, B)$ of clones equals $\{0\}\cup [q]$ with corresponding $1\times (q+1)$ proportion matrix $U_2(A, B) = [u^{(2)}_{1,j}]$ where $u^{(2)}_{1,j} = 1/q$ for all $j\in [q]$, and $u^{(2)}_{1,0} = 0$. The clone tree $T_1(A, B)$ is a star phylogeny rooted at $\Pi _1$-clone $i = 0$ with outgoing edges to each of the remaining $\Pi _1$-clones. Similarly, clone tree $T_2(A, B)$ is also a *star* phylogeny rooted at $\Pi _2$-clone $j = 0$ with outgoing edges to each of the remaining $\Pi _2$-clones. It is easy to verify that $U_1(A, B)$ and $U_2(A, B)$ are proportion matrices for $\Pi _1(A, B)$ and $\Pi _2(A, B)$, respectively. Clearly, this construction takes polynomial time. Figure 3c shows an example. The hardness follows from the following lemma whose proof is in Additional file 1: Section A.

#### **Lemma 2**

*Given proportions* $U_1(A, B)$ *and clone tree* $T_1$ *for clones* $\Pi _1(A, B) = \{0\}\cup [3q]$ *and proportions* $U_2(A, B)$ *and clone tree* $T_2$ *for clones* $\Pi _2(A, B) = \{0\}\cup [q]$*, there exists a set* $\Pi$ *of clones of size* $n = |\Pi | = 4q+1$*, clone tree* *T* *and proportion matrix* *U* *such that* *T* *is a refinement of* $T_1$ *and* $T_2$ *and* $J(U, U_1, U_2) = 0$ *if and only if there exists a solution of the* 3-partition *instance* (*A*, *B*).

## Methods

We introduce two mixed integer linear programming (MILP) formulations to solve the pci and the pcti problems. We implement these two formulations within the algorithm PACTION (PArsimonious Clone Tree integratION), which uses the MILP-solver Gurobi version 9.1. PACTION is available at https://github.com/elkebir-group/paction.

### Parsimonious Clone Integration

To solve the PCI problem, we introduce an MILP formulation composed of ${\mathcal {O}}(n_1 n_2 m)$ variables (including $O(n_1 n_2)$ binary variables) and ${\mathcal {O}}(n_1 n_2 m)$ constraints. We introduce binary variables $x_{i,j}\in \{0,1\}$ for each $\Pi _1$-clone $i\in [n_1]$ and $\Pi _2$-clone $j\in [n_2]$ that indicate if clone (*i*, *j*) belongs to $\Pi$. As such, the corresponding proportion of clone (*i*, *j*) in sample $p\in [m]$ is denoted by the continuous variable $u_{p,i,j}\in [0,1]$. In the following we define the constraints on these variables by first describing the constraints for consistency and next those for encoding the objective function.

*Consistency constraints* This first set of constraints ensure that proportion matrix *U* is consistent with proportion matrices $U_1$ and $U_2$. We begin by forcing $u_{p,i,j}$ to 0 if (*i*, *j*) is not a clone in the solution $\Pi$.

$$\begin{aligned} u_{p,i,j}&\le x_{i,j}&\forall p\in [m], i\in [n_1], j\in [n_2]. \end{aligned}$$

These above constraints allow us to model consistency of the solution *U* with input proportions $U_1=[u^{(1)}_{p,i}]$ and $U_2=[u^{(2)}_{p,j}]$ as follows.

$$\begin{aligned} \sum _{j=1}^{n_2} u_{p,i,j}&= u^{(1)}_{p,i}&\forall p\in [m], i\in [n_1], \\ \sum _{i=1}^{n_1} u_{p,i,j}&= u^{(2)}_{p,j}&\forall p\in [m], j\in [n_2]. \end{aligned}$$

Note that these two sets of constraints imply that $\sum _{i=1}^{n_1}\sum _{j=1}^{n_2} u_{p,i,j} = 1$ for all $p\in [m]$.

*Objective function* We minimize the total number of clones in the set $\Pi$ by minimizing the following objective function.

$$\begin{aligned} \min \sum _{i=1}^{n_1}\sum _{j=1}^{n_2} x_{i,j}. \end{aligned}$$

### Parsimonious Clone Tree Integration

To solve the PCTI problem, we introduce an MILP formulation composed of ${\mathcal {O}}(n_1 n_2 m)$ variables (including $O(n_1 n_2)$ binary variables) and ${\mathcal {O}}(n_1 n_2 m)$ constraints. Similarly to the pci MILP, we introduce binary variables $x_{i,j}\in \{0,1\}$ for $i\in [n_1]$ and $j\in [n_2]$ that indicate if clone (*i*, *j*) belongs to $\Pi$. As such, the corresponding proportion of clone (*i*, *j*) in sample $p\in [m]$ is denoted by the continuous variable $u_{p,i,j}\in [0,1]$. We introduce constraints to model the error $J(U,U_1,U_2)$ used in the objective function, as well constraints to enforce that *U* is a proportion matrix, and finally constraints to enforce that *T* is a refinement of $T_1$ and $T_2$.

*Correction constraints* Unlike the pci problem, the proportion matrix *U* need not be consistent with proportion matrices $U_1$ and $U_2$. We introduce continuous variables $c^{(1)}_{p,i}\in [0,1]$ for $p\in [m], i\in [n_1]$ and $c^{(2)}_{p,j}\in [0,1]$ for $p\in [m], j\in [n_2]$ to model the entry-wise absolute differences, i.e., $c^{(1)}_{p,i} = |\sum _{j=1}^{n_2} u_{p,i,j} - u^{(1)}_{p,i}|$ and $c^{(2)}_{p,j} = |\sum _{j=1}^{n_2} u_{p,i,j} - u^{(2)}_{p,j}|$. We do so with the following constraints.

$$\begin{aligned} c^{(1)}_{p,i}&\ge \sum _{j=1}^{n_2} u_{p,i,j} - u^{(1)}_{p,i}&\forall p\in [m], i\in [n_1], \\ c^{(1)}_{p,i}&\ge u^{(1)}_{p,i} - \sum _{j=1}^{n_2} u_{p,i,j}&\forall p\in [m], i\in [n_1],\\ c^{(2)}_{p,j}&\ge \sum _{i=1}^{n_1} u_{p,i,j} - u^{(2)}_{p,j}&\forall p\in [m], j\in [n_2],\\ c^{(2)}_{p,j}&\ge u^{(2)}_{p,j} - \sum _{i=1}^{n_1} u_{p,i,j}&\forall p\in [m], j\in [n_2]. \end{aligned}$$

*Proportion matrix constraints* To model that our output matrix *U* is a proportion matrix, we begin by ensuring that $u_{p,i,j} = 0$ with $x_{i,j} = 0$, i.e., the proportion of clone (*i*, *j*) is zero when it is not part of the solution $\Pi$ with the following constraints.

$$\begin{aligned} u_{p.i,j}&\le x_{i,j}&\forall p\in [m], i\in [n_1], j\in [n_2]. \end{aligned}$$

Next, we ensure that matrix *U* is a valid proportion matrix by enforcing that the proportions of the clones in each sample sum to 1.

$$\begin{aligned} \sum _{i=1}^{n_1}\sum _{j=1}^{n_2} u_{p,i,j}&= 1&\forall p\in [m]. \end{aligned}$$

*Refinement constraints* We introduce constraints that ensure that the clone tree *T* is a refinement of the clone trees $T_1$ and $T_2$. Following condition (iii) in Definition 3, we require that for each clone $(i,j) \ne (r(T_1),r(T_2))$ there only two possible parents, i.e., either $(i', j)$ or $(i, j')$ where $(i', i)\in E(T_1)$ and $(j', j)\in E(T_2)$. We model the first case with continuous variables $z^{(1)}_{(i,i'), j}\in [0,1]$ and the second case with continuous variables $z^{(2)}_{i,(j,j')}$. More specifically, we model the products $z^{(1)}_{(i,i'), j} = x_{i,j} x_{i', j}$ and $z^{(2)}_{i,(j,j')} = x_{i,j} x_{i,j'}$ with the following constraints.

$$\begin{aligned} z^{(1)}_{(i,i'), j}&\le x_{i,j}&\forall (i,i')\in E(T_1), j\in [n_2],\\ z^{(1)}_{(i,i'), j}&\le x_{i',j}&\forall (i,i')\in E(T_1), j\in [n_2],\\ z^{(1)}_{(i,i'), j}&\ge x_{i,j} + x_{i',j} - 1&\forall (i,i')\in E(T_1), j\in [n_2].\\ z^{(2)}_{i,(j,j')}&\le x_{i,j}&\forall i\in [n_1], (j,j')\in E(T_2),\\ z^{(2)}_{i,(j,j')}&\le x_{i,j'}&\forall i\in [n_1], (j,j')\in E(T_2),\\ z^{(2)}_{i,(j,j')}&\ge x_{i,j} + x_{i,j'} - 1&\forall i\in [n_1], (j,j')\in E(T_2). \end{aligned}$$

We now enforce conditions (i) and (ii) in Definition 3 as follows.

$$\begin{aligned} \sum _{j = 1}^{n_2} z^{(1)}_{(i,i'), j}&= 1&\forall (i,i')\in E(T_1), \\ \sum _{i = 1}^{n_1} z^{(2)}_{i, (j,j')}&= 1&\forall (j,j')\in E(T_2). \end{aligned}$$

*Objective function* Our goal is to minimize the difference between projections of proportion matrix *U* with $U_1$ and $U_2$. To that end, we minimize the following objective function

$$\begin{aligned} \min \sum _{p=1}^{m}\sum _{i=1}^{n_1} c^{(1)}_{p,i} + \sum _{p=1}^{m}\sum _{j=1}^{n_2} c^{(2)}_{p,j}. \end{aligned}$$

We provide the full MILP for reference in Additional file 1: Section D.

## Results

### Simulations

We perform simulations to investigate the performance of PACTION when solving the pci and pcti problems under different simulation regimes.

*Setup* Given numbers $n_1, n_2$ of clones, number *m* of samples and noise parameter $h\in [0,1]$, we use a three-step procedure to simulate a set $\Pi$ of $n = n_1 + n_2$ clones whose SNV and CNA evolution is described by a clone tree *T* and with clone proportions *U* on *m* samples. From *T* and *U*, we obtain input trees $T_1$ and $T_2$ as well as input proportion matrices $U_1$ and $U_2$ subject to additional noise *h*. We detail the three steps in the following.

First, we use an approach based on growing random networks [36] to simulate *T*: starting from the root vertex (representing the normal clone (1, 1)) *T*’s topology is built by iteratively adding descendant vertices, choosing each parent uniformly at random. Specifically, we label each edge with a single event from either the first set $\{2,\ldots ,n_1\}$ or second set $\{2,\ldots ,n_2\}$ of features. Thus, the overall clones $\Pi$ are obtained by labeling all vertices with a depth-first traversal. Second, we obtain the clone trees $T_1$ and $T_2$ by collapsing vertices of *T* corresponding to identical $\Pi _1$-clones and collapsing vertices of *T* corresponding to identical $\Pi _2$-clones, respectively. Third, the proportions *U* of the $\Pi$-clones in each sample are simulated by using a Dirichlet distribution with all concentration parameters equal to 1, similarly to previous methods [9, 27]. Proportions $U_1$ and $U_2$ are thus obtained following the consistency condition (Definition 2). Furthermore, we introduce noise in these two proportion matrices by mixing in a second draw from the same Dirichlet distribution using the parameter $h\in [0,1]$—a value of $h=0$ indicates the absence of noise. Details are in Additional file 1: Section E.

We ran PACTION in both PCI and PCTI mode on 360 simulated instances that we obtained by generating 10 instances for each combination of varying parameters. Matching numbers observed in recent cancer genomics studies [15, 23, 24], we varied the numbers $n_1\in \{3,5,8\}$ and $n_2\in \{3,5,8\}$ of clones, the number $m\in \{1,2,5\}$ of samples and noise level $h\in \{0, 0.05, 0.1, 0.15\}$. Note that both proportions $U_1,U_2$ and the simulated trees $T_1,T_2$ are taken in input in PCTI mode, while only proportions $U_1,U_2$ are considered in PCI mode.

**Fig. 4:**
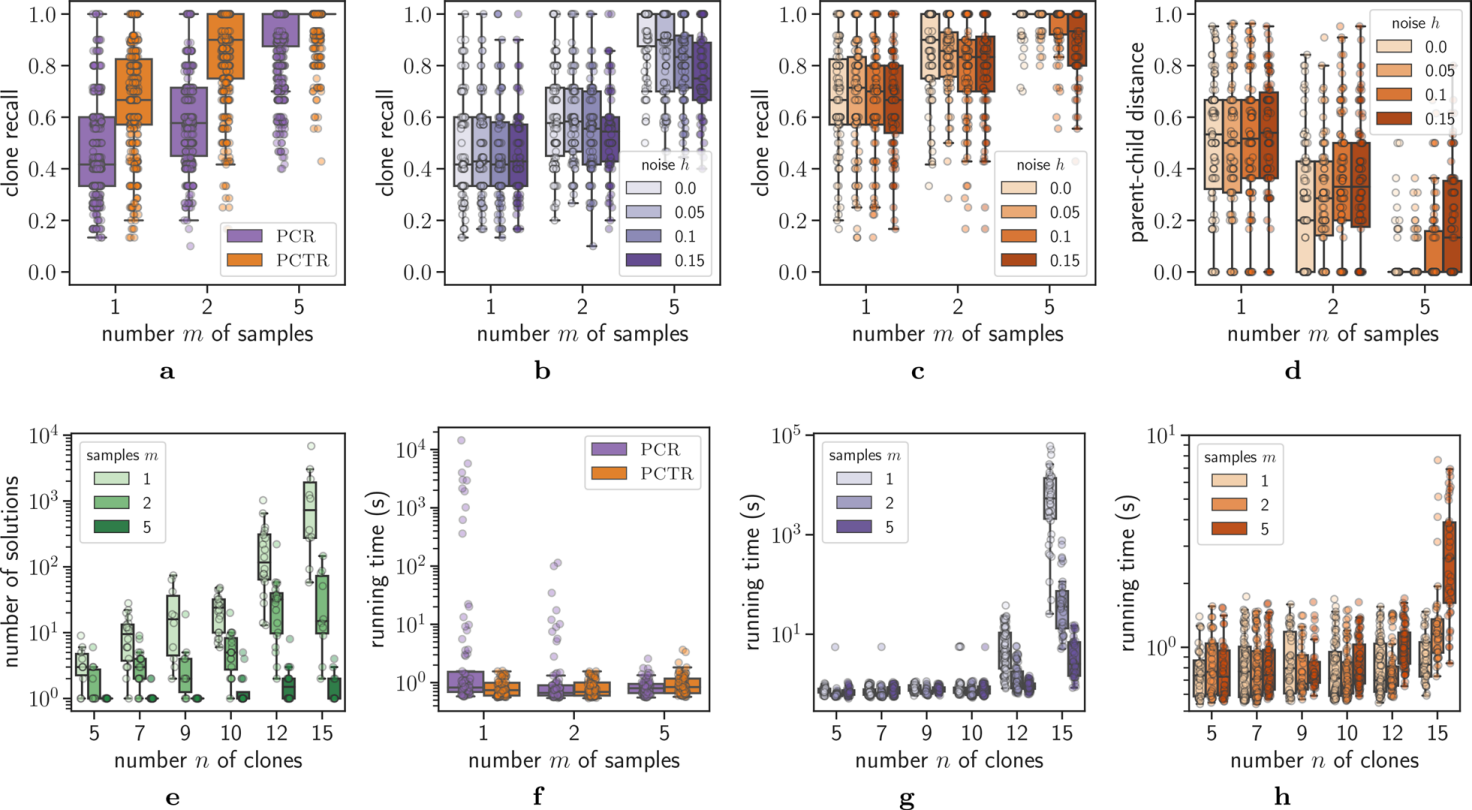
Simulations show that PACTION quickly and accurately reconstructs comprehensive clonal architectures. **a** Clone recall of PACTION in the pci and pcti mode for simulation instances with increasing number *m* of samples. Clone recall of PACTION in **b** pci mode and **c** pcti mode for different noise levels *h* and number *m* of samples. **d** Parent-child distance between the clone tree in the ground truth and the solution of PACTION in the pcti mode for simulation instances with increasing number *m* of samples. **e** Number of solutions to the error-free version of the pcti problem (with additional constraint of $J(U, U_1, U_2) = 0$) by SPRUCE (Additional file 1: Section C) for increasing number *n* of clones. **f** Running time of PACTION in the pci and pcti modes for simulation instances with increasing number *m* of samples. Running time of PACTION in **g** pci mode and **h** pcti mode for simulation instances with increasing number *n* of clones and number *m* of samples

*Results* We measure the performance of PACTION based on recall, which is the fraction of ground truth clones that are predicted by our method, i.e., the *clone recall* equals $|\Pi \cap \Pi ^*|/|\Pi ^*|$ where $\Pi$ is the set of clones inferred by PACTION and $\Pi ^*$ are the ground truth clones. As expected, PACTION in pcti mode leverages additional information from the clone trees $T_1$ and $T_2$ and thus resulted in higher recall compared to pci mode (Fig. 4a). Interestingly, recall increased with increasing number *m* of samples, as each additional samples provides additional constraints regarding consistency of the output clone proportions. Breaking down the clone recall by noise level *h*, we found that performance decreased with increasing noise levels in both pci mode (Fig. 4b) as well as pcti mode (Fig. 4c). However, we found that the pcti solver better handles increasing noise levels *h*, with a medial clone recall of 1 for noise level $h = 0$ as well as $h = 0.05$ when number *m* of samples is 5 (Fig. 4c and Additional file 1: Fig. S1).

Next, we investigated how well PACTION in pcti mode infers ground truth clone trees $T^*$. To that end, we computed the parent-child distance [37] between the predicted clone tree *T* and the clone tree $T^*$ in the ground truth. Specifically, the *parent-child distance* equals the ratio between the size $|E(T)\bigtriangleup E(T^*)|$ of the symmetric difference of the edge sets by the size $|E(T)\cup E(T^*)|$ of the union of edge sets. We observed that the clone tree distance is inversely correlated with the clone recall and when the clone recall is 1, the predicted clone tree matches the ground truth perfectly (Fig. 4d). Indeed, we observed that performance increases with increasing number *m* of samples, e.g., for $m=5$ samples the median parent-child distance is 0 for noise levels $h\in \{0,0.05,0.1\}$ indicating that in the majority of these instances PACTION perfectly inferred ground truth trees. The reason why performance drops for decreasing number of samples is because the number of solutions increases with decreasing number of samples (Fig. 4e). We used the correspondence between the pcti problem (subject to the constraint that $J(U,U_1,U_2)=0$, i.e., the proportions are error-free) and the perfect phylogeny mixture problem solved by SPRUCE [21] to enumerate all solutions for $h=0$ instances (details in Additional file 1: Section C). For instances with a large number of optimal solutions, the pcti problem and consequently the MILP lacks additional constraints to disambiguate between solutions, thus sometimes reporting solutions that do not match the ground truth.

Finally, we investigated the running times of PACTION in pci and pcti modes. Overall, the running times in pci mode (median of 0.79 s and mean of 385.52 s) were larger than pcti mode (median of 0.77 s and mean of 0.95 s), likely due to the tree constraints providing more guidance for the MILP solver (Additional file 1: Table S1). Interestingly, while running time decreased with increasing number *m* of samples in pci mode, the opposite is true in pcti mode. The reason is that in pcti mode the MILP is often solved in the first iteration prior to branching, where the running time of solving the linear programming relaxation will depend on the size of the formulation, which in turn depends on *m*. However, in pci mode, the solver requires branching, and here additional constraints due to more samples will provide stronger bounds that will lead to more pruning and reduction in overall running time.

In summary, our simulations demonstrate that PACTION is able to quickly and accurately reconstruct ground truth clonal architectures under varying noise levels *h*, especially when the number *m* is large and when run in pcti mode.

### Metastatic prostate cancer

In this study, we analyze whole-genome sequencing data from 49 tumor samples from 10 metastatic prostate cancer patients [23]. In a previous analysis of this data, Gundem et al. [23] identified SNV clones and reconstructed the SNV clone tree for each of the 10 patients. To further investigate the role of CNAs on tumor evolution, the authors annotated the SNV clone trees with CNA events in a post hoc analysis by manually comparing and matching frequencies of SNVs and CNAs. However, this approach does not allow us to identify tumor clones that are only distinguished by different CNAs and have the same SNVs. Therefore, there is no information about CNA-only driven tumor clones nor information about the ordering of the CNA events and the SNV events on the same edge of the tree. Such information is crucial to understand cancer progression [38] and is the subject of numerous studies [39–41]. Therefore, we investigated whether we can use PACTION to provide a more comprehensive analysis of these tumor clonal compositions by jointly considering SNVs and CNAs.

We applied PACTION to previously inferred SNV and CNA clone proportions. First, we used the SNV clone proportions as well as the SNV clone tree $T_1$ inferred for each patient by Gundem et al. [23]. Note that each edge of the SNV tree represents a cluster of SNV mutations. As such, we computed the SNV clone proportions $U_1$ using the published cancer cell fractions of SNVs (details in Additional file 1: Section F). Second, we used the CNA clones obtained from a previous copy-number analysis [15] of the same patients. Since this previous analysis does not provide CNA clone trees, we enumerated all possible binary trees [42] with the CNA clones as the leaves and independently ran PACTION in pcti mode with each tree as input. We then selected the CNA clone tree with the smallest correction $J(U, U_1, U_2)$, which for each patient was unique. results. Overall, we ultimately obtained SNV trees with $n_1 \in \{5, \ldots , 16\}$ clones and CNA trees with $n_2 \in \{4, \ldots , 8\}$ clones across $m \in \{2, \ldots , 10\}$ samples (Additional file 1: Table S2).

**Fig. 5:**
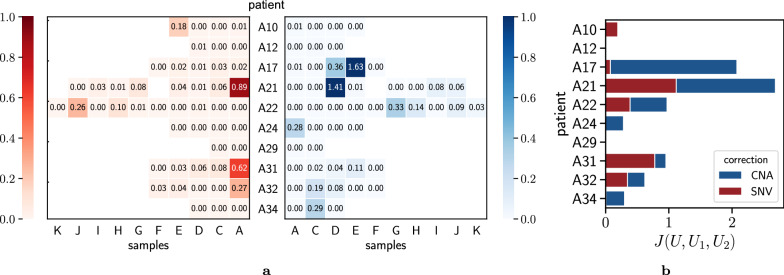
Overview of PACTION results on samples from 10 metastatic prostate cancer patients [23]. **a** The corrections made by PACTION to the SNV and CNA clone proportions in the samples from each of the 10 patients. **b** The total correction made to clone proportions $J(U, U_1, U_2)$ in samples from each patient

In all patients but A29, we found that one cannot integrate independently-inferred SNV and CNA clone trees without additional corrections to the clone proportions. Importantly, this observation highlights that the clone proportions inferred by existing methods are generally characterized by errors (Fig. 5a). As previously demonstrated in our simulation study, PACTION, however, reliably handles the presence of noise, enabling the inference of the complete clonal composition and tumor evolution with limited corrections for all patients. Specifically, the corrections applied by PACTION were limited to only a few samples per patient, potentially indicating sample-specific errors in previous analysis or samples with higher levels of noise. Importantly, we also observed that corrections were uniformly needed for both SNV and CNA clone proportions (Fig. 5). This important observation highlights that both features are generally characterized by errors and, therefore, one cannot simply leave one feature fixed and use it to reconcile the other feature, as done previously [23].

**Fig. 6:**
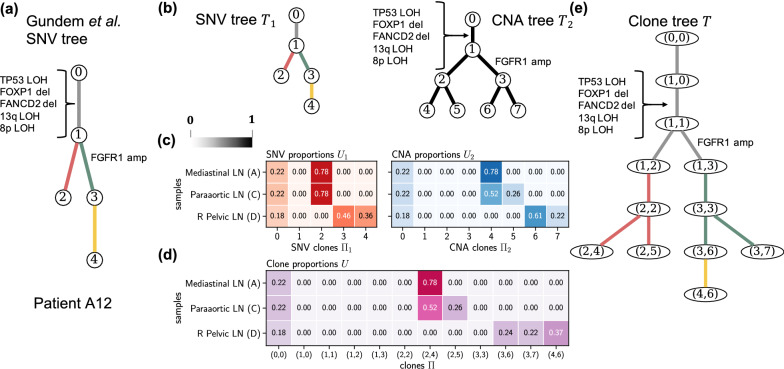
PACTION results for patient A12. **a** The SNV clone tree reported by Gundem et al. [23] where the authors manually annotated edges with CNA events. **b** SNV clone tree $T_1$ and CNA clone tree $T_2$ describing the evolution of the SNV clones $\Pi _1$ and CNA clones $\Pi _2$ in the tumor samples of patient A12, respectively. **c** Proportions $U_1$ of SNV clones $\Pi _1$ and proportions $U_2$ of CNA clones $\Pi _2$ in the four samples of patient A12. **d** Proportions *U* of tumor clones $\Pi$ in the four samples of patient A12 inferred by PACTION. **e** Integrated clone tree *T* inferred by PACTION. amp: amplification, del: deletion, LOH: loss of heterozygosity

Notably, we found that the integrated clone trees inferred by PACTION reveal additional branching events that were previously missed. As an example, in patient A12, Gundem et al. [23] inferred an SNV clone tree with five clones and annotated this tree with five clonal CNA events, including loss-of-heterozygosity (LOH) of gene TP53 and chromosomes 8p and 13q, as well as deletions of genes FOXP1 and FANCD2 (gray edge in Fig. 6a). The tree also contains a single subclonal CNA event, amplification of gene FGFR1 (green edge in Fig. 6a). When using PACTION to analyze the previously-inferred SNV and CNA clone proportions, we reconstructed a integrated clone tree with higher resolution. In fact, PACTION reconstructed a more refined clone tree with 12 clones while only applying modest corrections to the input clone proportions (Fig. 5a). Similarly to the published tree, PACTION’s inferred clone tree contains a trunk with the same four clonal CNA events. However, PACTION’s tree contains additional branching events that are absent in the published SNV tree. Specifically, we observed that two SNV clones in the published tree (i.e., 2 and 3) were split into multiple clones in PACTION’s refined tree (i.e., (2, 2), (2, 4), and (2, 5) for SNV clone 2, and (3, 3), (3, 6), and (3, 7) for SNV clone 3). Importantly, a subset of these refined clones are present at large proportions in the sequenced samples (Fig. 6d), thus showing that PACTION enables a more fine-grained analysis of current sequencing data.

**Fig. 7:**
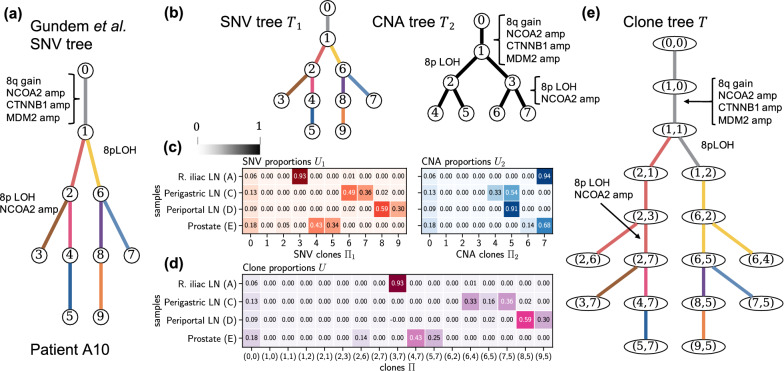
PACTION results for patient A10. **a** The SNV clone tree reported by Gundem et al. [23] where the authors manually annotated edges with CNA events. **b** SNV clone tree $T_1$ and CNA clone tree $T_2$ describing the evolution of the SNV clones $\Pi _1$ and CNA clones $\Pi _2$ in the tumor samples of patient A12, respectively. **c** Proportions $U_1$ of SNV clones $\Pi _1$ and proportions $U_2$ of CNA clones $\Pi _2$ in the four samples of patient A10. **d** Proportions *U* of tumor clones $\Pi$ in the four samples of patient A10 inferred by PACTION. **e** Integrated clone tree *T* inferred by PACTION. amp: amplification, LOH: loss of heterozygosity

Finally, we found that the more refined clone trees inferred by PACTION also reveal novel insights about the relative temporal ordering of SNVs and CNAs. This phenomenon is particularly interesting in patient A10 (Fig. 7a), for which PACTION inferred a clone tree with 17 clones and relatively high corrections to the previous SNV clone proportions (Fig. 7b–d). PACTION’s tree recapitulates the same four clonal CNAs identified in the previous tree, including gain of chromosome 8q and amplifications of genes NCOA2, CTNNB1 and MDM2 (gray edge in Fig. 7a). Importantly, PACTION’s tree also recapitulates subclonal CNA events as in the previous tree but further revealed that these CNA events precede the SNV events placed on the same edges in the published SNV clone tree (Fig. 7e). More specifically, PACTION revealed that LOH of chromosome 8p and amplification of gene NCOA2 occur on the edge from clone (2, 3) to (2, 7) which precedes the SNV cluster represented by the edge from clone (2, 7) to (3, 7). Similarly, PACTION revealed that LOH of chromosome 8p occurs on the edge from clone (1, 1) to (1, 2) which precedes the SNV cluster represented by the edge from clone (1, 2) to (6, 2).

In summary, we demonstrated on metastatic prostate cancer patients that PACTION is able to resolve the temporal ordering of mutations and reveal branching events that are either unclear or hidden when the SNV tree or the CNA tree are considered in isolation.

## Discussion

In this paper, we introduced PACTION, a new algorithm that infers comprehensive tumor clonal compositions by integrating the clones proportions of both SNVs and CNAs that are inferred by existing methods. Our algorithm can additionally leverage SNV and CNA clone trees reconstructed by existing methods to obtain a refined tumor clone tree and correct potential errors in the input proportions. We formulated two problems, the pci problem to infer the clones and their proportions, and the pcti problem to additionally infer tumor clone trees with both SNVs and CNAs. We showed that both problems are NP-hard and can be solved exactly by PACTION using two mixed inter linear programming formulations. We demonstrated the performance of PACTION on simulations, showing that our method accurately reconciles clone trees, reliably handles errors in clone proportions, and scales to practical input sizes. Finally, we applied our method to whole-genome sequencing data from 10 metastatic prostate cancer patients [23], obtaining a higher resolution view of tumor evolution than previously reported.

In addition to the contributions of this study, we foresee four major avenues for future research. First, building upon the established relationship of the error-free pcti and the cladistic multi-state perfect phylogeny deconvolution problems (Additional file 1: Section C), we can adapt the existing method SPRUCE [21] to enumerate all possible solution of the pcti problem in the presence of errors in the input proportions. Second, PACTION can be extended to account for uncertainty in the input clone trees and quantify its effect on the solution space. One way of incorporating the uncertainty in the input clone trees, is to consider a set of possible clone trees for each feature instead of a single input tree, choosing the best tree that leads to the most parsimonious solution. Moreover, we plan to adapt the pci and pcti to incorporate probabilistic models that account for uncertainty in the estimated clone proportions. Third, the pci and pcti problems can be generalized to integrate more than two features. For instance, in addition to SNVs and CNAs, tumor cells may be partitioned into clones based on RNA expression or DNA methylation profiles. Finally, a likelihood-based objective function could be used to incorporate a joint evolutionary model for SNVs and CNAs [43].

## Supplementary Information

**Additional file 1. **Supplementary proofs for lemmas and theorems in the main text, a section showing the equivalence of the PCTI problem and the Multi-state Cladistic Perfect Phylogeny Mixture Deconvolution problem, detailed MILP formulation for the PCTI problem, simulation and real data processing details, and 1 figure and 2 tables describing additional results.

## Data Availability

PACTION is available at https://github.com/elkebir-group/paction.
