# Supplementary material for: Parsimonious Clone Tree Integration in cancer
Source: Algorithms Mol Biol. 2022 Mar 14;17:3. doi: 10.1186/s13015-022-00209-9 (PMC8919608; doi:10.1186/s13015-022-00209-9)
Supplement: Supplementary file 1 — Additional file 1. Supplementary proofs for lemmas and theorems in the main text, a section showing the equivalence of the PCTI problem and the Multi-state Cladistic Perfect Phylogeny Mixture Deconvolution problem, detailed MILP formulation for the PCTI problem, simulation and real data processing details, and 1 figure and 2 tables describing additional results. [file 13015_2022_209_MOESM1_ESM.pdf]

Supplementary Material for "Parsimonious Clone Tree Integration in Cancer"

Palash Sashittal[1], Simone Zaccaria[2,3], and Mohammed El-Kebir[1,4*]

[1]Dept. of Computer Science, University of Illinois Urbana-Champaign, IL 61801

[2]Computational Cancer Genomics Research Group, University College London Cancer Institute, London, UK

[3]Cancer Research UK Lung Cancer Centre of Excellence, University College London Cancer Institute, London, UK

[4]Cancer Center at Illinois, University of Illinois Urbana-Champaign, Urbana, IL

[*]Correspondence: melkebir@illinois.edu

Contents

A Supplementary Proofs 2

B Checking Consistency of a Proportion Matrix for a Given Instance of the PCI Problem 5

C Equivalence of the PCTI and the Multi-state Cladistic Perfect Phylogeny Mixture Deconvolution Problems 6

D MILP formulation for the PCTI problem 8

E Simulation Details 9

F Computation of SNV Clone Proportions 10

G Supplementary Figures and Tables 11

A Supplementary Proofs

(Main Text) Lemma 1. Given proportions $U_1(A,B)$ for clones $\Pi_1(A,B) = [3q]$ and proportions $U_2(A,B)$ for clones $\Pi_2(A,B) = [q]$, there exists a set Π of clones of size $n = |\Pi| \leq 3q$ with proportions U that are consistent with $U_1(A,B)$ and $U_2(A,B)$ if and only if there exists a solution to the 3-PARTITION instance (A,B).

Proof. ($\Rightarrow$) Let clones Π and proportion matrix U be a solution to the PCI problem instance $(\Pi_1(A,B), U_1(A,B), \Pi_2(A,B), U_2(A,B))$. By the premise, we have that $n = |\Pi| \leq 3q$. Note that since $n_1 = |\Pi_1| = 3q > q = |\Pi_2| = n_2$ and $u^{(1)}_{1,i} > 0$ for all $i \in [3q]$, by Observation 1, we have $n = |\Pi| \geq 3q$. Putting this together with the upper bound $n = |\Pi| \leq 3q$, obtained from the premise, we have that the PCI solution has $n = 3q$ clones. Note that since $|\Pi_1| = |\Pi|$, for every $i \in [3q]$, there is a unique $j \in [q]$ such that $(i,j) \in \Pi$. Since U is consistent with U_1 and u_2, we have that

$$u_{1,(i,j)} = u^{(1)}_{1,i}. \tag{1}$$

We claim that the solution to the 3-PARTITION problem instance (A,B) is given by the function $\sigma(i) = j$ where $(i,j) \in \Pi$, for each $i \in [3q]$.

We show that σ defined above satisfies Equation (1) in the main text. Recall that $\pi_1((i,j)) = i$ and $\pi_2((i,j)) = j$. For any $j \in [q]$, we have

$$\begin{aligned}\sum_{i\in\sigma^{-1}(j)} a_i &= \sum_{(i,j')\in\Pi:\pi_2((i,j'))=j} a_i \\ &= \sum_{(i,j')\in\Pi:\pi_2((i,j'))=j} Bqu^{(1)}_{1,s} \\ &= \sum_{(i,j')\in\Pi:\pi_2((i,j'))=j} Bqu_{1,(i,j)} \\ &= Bqu^{(2)}_{1,j} = B,\end{aligned}$$

where the second equality follows from construction with $u^{(1)}_{1,i} = a_i/(Bq)$, the third equality uses Equation (1), the fourth equality uses consistency of proportion matrix U with respect to U_2 given projection function π_2 and the fifth equality uses the construction $u^{(2)}_{1,j} = 1/q$.

($\Leftarrow$) Let $\sigma : [3q] \to [q]$ be a solution to the 3-PARTITION problem instance (A,B). We claim that $\Pi = \{(i,\sigma(i)) : i \in [3q]\}$ with $n = |\Pi| = 3q$ clones and $1 \times n$ proportion matrix $U = [u_{1,(i,\sigma(i))}]$ where $u_{1,(i,\sigma(i))} = u^{(1)}_{1,i} = a_i/(Bq)$ is a solution to the corresponding PCI problem.

To see why, recall that $\pi_1((i,\sigma(i))) = s$ and $\pi_2((i,\sigma(i))) = \sigma(i)$. Given these projection functions, we need to show that $U$is consistent with $U_1(A,B)$ and $U_2(A,B)$. The consistency with respect to the

Π_1-clones is trivial as for each $i \in \Pi_1 = [3q]$ there exists exactly one pair $(i, j) \in \Pi$, i.e., the pair (i, j) where $j = \sigma(i)$, with proportion $u_{1,(i,\sigma(i))} = u^{(1)}_{1,i}$. To see the consistency with respect to the Π_2-clones, consider for any $j \in \Pi_2 = [q]$,

$$\sum_{(i,\sigma(i)):\sigma(i)=j} u_{1,(i,\sigma(i))} = \sum_{i\in\sigma^{-1}(j)} u^{(1)}_{1,i} = \sum_{i\in\sigma^{-1}(j)} \frac{a_i}{Bq} = \frac{1}{Bq}B = \frac{1}{q},$$

where the second to last equality uses Equation (1). Since $u^{(2)}_{1,j} = 1/q$ for all $j \in [q]$, we have

$$\sum_{(i,\sigma(i)):\sigma(i)=j} u_{1,(i,\sigma(i))} = u^{(2)}_{1,j}, \quad \forall j \in [q],$$

which is the required condition for consistency. □

(Main Text) Lemma 2. Given proportions $U_1(A, B)$ and clone tree T_1 for clones $\Pi_1(A, B) = \{0\} \cup [3q]$ and proportions $U_2(A, B)$ and clone tree T_2 for clones $\Pi_2(A, B) = \{0\} \cup [q]$, there exists a set Π of clones of size $n = |\Pi| = 4q + 1$, clone tree T and proportion matrix U such that T is a refinement of T_1 and T_2 and $J(U, U_1, U_2) = 0$ if and only if there exists a solution of the 3-PARTITION instance (A, B).

Proof. ($\Rightarrow$) Let clones Π, clone tree T and proportion matrix U be a solution to the PCTI problem instance $(\Pi_1(A, B), T_1(A, B), U_1(A, B), \Pi_2(A, B), T_2(A, B), U_2(A, B))$. By the premise, we have that $J(U, U_1, U_2) = 0$, which implies that U is consistent with U_1 and U_2.

Note that by Observation 7 in the main text, since $r(T_1) = 0$ and $r(T_2) = 0$, we have $(0, 0) \in \Pi$ and $r(T) = (0, 0)$. Recall that $\pi_1 : \Pi \to \Pi_1$ maps each clone in Π to its corresponding Π_1-clone. We claim that this function is a bijection for this construction. That is, $|\pi_1^{-1}(i)| = 1$ for all $i \in [3q]$. Clearly $|\pi_1^{-1}(i)| \geq 1$ by Definition 3 condition (i) and Observation 7 in the main text. As for why $|\pi_1^{-1}(i)| \leq 1$, assume for a contradiction, there exists an $i \in [3q]$ such that $\{j, j'\} \subseteq \pi_1^{-1}(i)$, i.e. $(i, j), (i, j') \in \Pi$ for two distinct $j, j' \in \Pi_2$. Since $(i, j) \in \Pi$, by Observation 8 in the main text either $((0, j), (i, j)) \in E(T)$ or $((i, 0), (i, j)) \in E(T)$. Similarly since $(i, j') \in \Pi$, by Observation 8 in the main text, either $((0, j'), (i, j')) \in E(T)$ or $((i, 0), (i, j')) \in E(T)$. Putting these two conditions together, either $\{((0, j), (i, j)), ((0, j'), (i, j'))\} \subseteq E(T)$ or $\{((i, 0), (i, j)), ((i, 0), (i, j'))\} \in E(T)$. We analyze these two cases as follows.

- **Case 1**: Consider $\{((0, j), (i, j)), ((0, j'), (i, j'))\} \subseteq E(T)$. This violates condition (i) in the Definition 3 in the main text of T being a refinement of T_1 and T_2.

- **Case 2**: Consider $\{((i, 0), (i, j)), ((i, 0), (i, j'))\} \in E(T)$. Note that the proportion $u^{(1)}_{1,i}$ of each clone $i \in \Pi_1 \setminus \{0\}$ occurs within the open interval $(1/4q, 1/2q)$ and therefore $u^{(1)}_{1,i} < 1/q$. Since the proportion $u^{(2)}_{1,j} = 1/q$ for any clone $j \in \Pi_2 \setminus \{0\}$, we have $|\pi_2^{-1}(j)| > 1$. Let $i' \in \pi_2^{-1}(j)$ such that $i \neq i'$.

Since $(i', j) \in \Pi$, by Observation 8 in the main text, either $((i', 0), (i', j)) \in E(T)$ or $((0, j), (i', j)) \in E(T)$. If $((i', 0), (i', j)) \in E(T)$, since by premise $\{((i, 0), (i, j)), ((i, 0), (i, j'))\} \in E(T)$, it will violate condition (ii) in the Definition 3 in the main text of T being refinement of T_1 and T_2. Alternatively, $((0, j), (i', j)) \in E(T)$ implies that $(0, j) \in \Pi$, which by condition (iii) in Definition 3 in the main text entails $((0, 0), (0, j)) \in E(T)$. However, since $((i, 0), (i, j)) \in E(T)$, this would violate condition (ii) in Definition 3 in the main text.

Therefore, $|\pi_1^{-1}(i)| = 1$ for $i \in [3q]$. Also, since $u_{1,0}^{(2)} = 0$ and since the proportion matrix U is consistent with U_1 and U_2, we have $\pi_1^{-1}(i) = j \in [q]$ for each $i \in [3q]$. We claim that the solution of the 3-PARTITION problem instance (A, B) is $\sigma(i) = j$ for $(i, j) \in \Pi$, for each $i \in [3q]$. We show that σ defined above satisfies Equation (1) in the main text. Recall that $\pi_1((i, j)) = i$ and $\pi_2((i, j)) = j$. For any $j \in [q]$, we have

$$
\begin{aligned}
\sum_{i \in \sigma^{-1}(j)} a_i &= \sum_{(i,j') \in \Pi : \pi_2((i,j')) = j} a_i \\
&= \sum_{(i,j') \in \Pi : \pi_2((i,j')) = j} Bq u_{1,s}^{(1)} \\
&= \sum_{(i,j') \in \Pi : \pi_2((i,j')) = j} Bq u_{1,(i,j)} \\
&= Bq u_{1,j}^{(2)} \\
&= Bq \frac{1}{q} \\
&= B,
\end{aligned}
$$

where the second equality follows from construction with $u_{1,i}^{(1)} = a_i/(Bq)$, the third equality uses Equation (1), the fourth equality uses consistency of proportion matrix U with respect to U_2 given projection function π_2 and the fifth equality uses the construction $u_{1,j}^{(2)} = 1/q$.

($\Leftarrow$) Let $\sigma : [3q] \to [q]$ be a solution to the 3-PARTITION problem instance (A, B). We claim that $\Pi = \{(0, j) : j \in [q] \cup \{0\}\} \cup \{(i, \sigma(i)) : i \in [3q]\}$ with $n = |\Pi| = 4q + 1$ clones, clone tree T with edges $E(T) = \{((0, 0), (0, j)) : j \in [q]\} \cup \{((0, j), (i, j)) : i \in \sigma^{-1}(j), j \in [q]\}$ and $1 \times (4q + 1)$ proportion matrix $U = [u_{1,(i,j)}]$ where $u_{1,(i,\sigma(i))} = u_{1,i}^{(1)} = a_i/(Bq)$ when $i \in [3q]$, $u_{1,(0,j)} = 0$ for $j \in [q] \cup \{0\}$ is a solution to the PCTI problem with $J(U, U_1, U_2) = 0$.

We first show that U is consistent with respect to U_1 and U_2 which is equivalent to the condition $J(U, U_1, U_2) = 0$. Recall the projection functions $\pi_1((i, j)) = i$ and $\pi_2((i, j)) = j$. We show consis-

tency with respect to U_1 as follows. For $i = 0$, since $\pi_1^{-1}(0) = [q] \cup \{0\}$, we have

$$\sum_{j \in \pi_1^{-1}(0)} u_{1,(0,j)} = \sum_{j=0} u_{1,(0,j)} = 0 = u_{1,0}^{(1)}.$$

For $i \in [3q]$, since $\pi_1^{-1}(i) = \sigma(i)$ we have,

$$\sum_{j \in \pi_1^{-1}(i)} u_{1,(i,j)} = u_{1,(i,\sigma(i))} = a_i/(Bq) = u_{1,i}^{(1)}.$$

We show consistency with respect to U_2 as follows. For $j = 0$, since $\pi_2^{-1}(0) = 0$, we have

$$\sum_{i \in \pi_2^{-1}(0)} u_{1,(i,0)} = u_{1,(0,0)} = 0 = u_{1,0}^{(2)}.$$

For $j \in [q]$, since $\pi_2^{-1}(j) = \sigma^{-1}(j)$, we have

$$\sum_{i \in \pi_2^{-1}(j)} u_{1,(i,j)} = \sum_{i \in \sigma^{-1}(j)} u_{1,(i,j)} = \sum_{i \in \sigma^{-1}(j)} a_i/(Bq) = B/(Bq) = 1/q = u_{1,j}^{(2)},$$

where the third equality uses the premise that σ is a solution of the 3-PARTITION problem instance (A, B).

Now we show that T is a refinement of T_1 and T_2. We address the three conditions in Definition 3 in the main text as follows.

- **Condition (i):** Recall that $E(T_1) = \{(0, i) : i \in [3q]\}$. For each edge $(0, i) \in E(T_1)$, we have a unique $j = \sigma(i) \in [q]$ such that $((0, j), (i, j)) \in E(T)$.

- **Condition (ii):** Recall that $E(T_2) = \{(0, j) : j \in [q]\}$. For each edge $(0, j) \in E(T_2)$, we have a unique $i = 0$ such that $((0, 0), (0, j)) \in E(T)$.

- **Condition (iii):** Recall that $E(T) = \{((0, 0), (0, j)) : j \in [q]\} \cup \{((0, j), (i, j)) : i \in \sigma^{-1}(j), j \in [q]\}$. For each edge $((0, 0), (0, j)) \in E(T), j \in [q]$, we have $(0, j) \in E(T_2)$ and for edge $((0, j), (i, j)) \in E(T), i \in \sigma^{-1}(j), j \in [q]$, we have $(0, i) \in E(T_1)$.

□

B Checking Consistency of a Proportion Matrix for a Given Instance of the PCI Problem

In this section, given an instance (Π_1, U_1, Π_2, U_2) of the PCI problem and a set $\Pi \subseteq \Pi_1 \times \Pi_2$, we describe the procedure to check if there exists a proportion matrix U that is consistent with given proportion matrix

U_1 for clones Π_1 and U_2 for clones Π_2 in polynomial time. We do this by reduction to the maximum flow problem.

Recall that maximum flow problem is as follows. Given a directed graph $G = (V, E)$ with a source $s \in V$ and sink $t \in V$ and capacities along every each described by $c : E \to \mathbb{R}^+$, find a flow $f : E \to \mathbb{R}$ that maximizes the total flow through the sink defined by

$$f_s = \sum_{e \in \delta^+(s)} f(e),$$

where $\delta^+(s)$ denotes the set of outgoing edges from s in G. Briefly, a function $f : E \to \mathbb{R}$ is a *flow* provided (i) f satisfies flow conservation, i.e. the sum of the flow on the incoming edges of each vertex (distinct from s and t) must equal the outgoing flow, the (ii) the value $f(e)$ of each edge e must be nonnegative and cannot exceed its capacity $c(e)$. The reader is referred to [1] for more details.

For a given instance (Π_1, U_1, Π_2, U_2) of the PCI problem, we construct m instances of the maximum flow problem with different capacity functions, but the same underlying graph $G = (V, E)$ constructed as follows. Recall that $\Pi = [n_1]$ and $\Pi_2 = [n_2]$. Let $V = \{s, t\} \cup \{v_1, \ldots, v_{n_1}\} \cup \{w_1, \ldots, w_{n_2}\} \cup \Pi_2$ and $E = \{(s, u_i) : \forall i \in \Pi_1\} \cup \{(u_i, w_j) : \forall (i, j) \in \Pi\} \cup \{(j, t) : \forall j \in \Pi_2\}$. The capacity function c_p, for p^{th} instance of the maximum flow problem is defined such that $c_p((i, j)) = 1, \forall (i, j) \in \Pi$, $c_p((s, i)) = u^{(1)}_{p,i}$ and $c_p(j, t) = u^{(2)}_{p,j}$. Clearly, the maximum possible flow through the source s in the pth instance is bounded from above by the total capacity of the edges in $\delta^+(s)$ given by $\sum_{i=1}^{n_1} u^{(1)}_{p,i} = 1$. In fact there exists a proportion matrix U that is consistent with given proportion matrix U_1 for clones Π_1 and U_2 for clones Π_2 if an only if the maximum flow is *equal* to 1 for all the m instances of maximum flow problem. In such a case, let f_p be the flow for the pth instance. The proportion matrix $U \in [0, 1]^{m \times |\Pi|}$ is given by $u_{p,(i,j)} = f_p(i, j)$. We refer to (Main Text) Figure 2b for an example.

C Equivalence of the PCTI and the Multi-state Cladistic Perfect Phylogeny Mixture Deconvolution Problems

In this section we show that the error-free version of the PCTI problem is equivalent to a special case of the previously posed Cladistic Multi-state Perfect Phylogeny Mixture Deconvolution problem [2]. To state the latter problem, we recall the definition of a multi-state perfect phylogeny from References [3, 4]. Note that we keep the same notations as in the original paper [2] and thus there will be some overlap in notation to the main text of this paper.

Definition 1 ([3, 4])**.** A rooted tree T is a *multi-state perfect phylogeny* on n characters provided that (1) each vertex is labeled by a *state vector* $\mathbf{a} \in \mathbb{N}^n$, which denotes the state for each character; (2) the root vertex of T has state 0 for each character; (3) vertices labeled with state i for character c form a connected subtree $T_{(c,i)}$ of T.

Let T be a multi-state perfect phylogeny on n characters. For each non-empty subtree $T_{(c,i)}$ for character-state pair (c, i), we denote the root vertex of this subtree by $v_{(c,i)}$. We note that $v_{(c,0)}$ for each character $c \in [n]$ equals the root vertex $r(T)$ of T. For two character-state pairs (c, i) and (d, j) that induce non-empty subtrees $T_{(c,i)}$ and $T_{(d,j)}$ we write $(c, i) \preceq_T (d, j)$ if vertex $v_{(c,i)}$ occurs on the unique path from the root vertex $r(T)$ to vertex $v_{(d,j)}$. Note $\preceq_T$ is a reflexive relation, i.e., it holds that $v_{(c,i)} \preceq_T v_{(c,i)}$ for all non-empty connected subtrees $T_{(c,i)}$ of T.

In the case of cladistic characters we are given a set $\mathcal{S} = \{S_1, \ldots, S_n\}$ of state trees for each character. The vertex set of a state tree S_c is $\{0, \ldots, k-1\}$, and the edges describe the relationships of the states of character c. Formally, we define a state tree as follows.

Definition 2. A rooted, vertex-labeled tree S on k vertices is a *state tree* provided (i) each vertex v_i is labeled uniquely by a state i from the set $\{0, \ldots, k-1\}$ and (ii) the root vertex $r(S)$ is labeled by state 0.

Similarly to above, we write $v_i \preceq_S v_j$ if vertex $v_i \in V(S)$ occurs on the unique path from the root vertex $r(S)$ to vertex $v_j \in V(S)$. This enables us to define consistency as follows.

Definition 3. A multi-state perfect phylogeny T is *consistent* with state trees $\mathcal{S} = \{S_1, \ldots, S_n\}$ provided $v_i \prec_{S_c} v_j$ if and only if $v_{(c,i)} \prec_T v_{(c,j)}$ for all characters $c \in [n]$ and states $i, j \in \mathbb{N}$.

Note that multiple character-state pairs may label the same edge in a multi-state perfect phylogeny. To prevent this from happening, we need to impose an additional constraints that ensure that each non-root vertex $v \neq r(T)$ of T corresponds to a unique character-state pair (c, i) that indicates the single change that happened on its incoming edge. Specifically, we call such constrained trees *complete multi-state perfect phylogenies consistent with* $\mathcal{S} = \{S_1, \ldots, S_n\}$.

Rather than observing such a tree, in practice, we observe an $m \times |V(S_c)|$ frequency matrix $F_c = [f^{(c)}_{p,i}]$ for each character $c \in [n]$, where entries $f^{(c)}_{p,i}$ indicate the fraction of cells in sample p having state i for character c.

Definition 4. An $m \times |V(S_c)|$ matrix $F_c = [f^{(c)}_{p,i}]$ is a *frequency matrix* for state tree $S_c \in \mathcal{S}$ provided (i) $f^{(c)}_{p,i} \geq 0$ for each sample p, and (ii) $\sum_{i=0}^{|V(S_c)|-1} f^{(c)}_{p,i} = 1$.

Given frequencies matrices $F_1, \ldots, F_n$ on m samples for n cladistic characters with state trees $\mathcal{S} = \{S_1, \ldots, S_n\}$, we seek a complete multi-state perfect phylogeny T consistent with $\mathcal{S}$ and an $m \times |V(T)|$ proportion matrix U (following Definition 1 in the main text) that explains the given frequency matrices. More formally, the problem is posed as follows [2].

Problem 1 (Cladistic Multi-state Perfect Phylogeny Deconvolution (CMPPD) [2])**.** Given frequency matrices $F_1, \ldots, F_n$ on m samples for state trees $\mathcal{S} = \{S_1, \ldots, S_n\}$, find a complete multi-state perfect phylogeny tree T consistent with $\mathcal{S}$ and an $m \times |V(T)|$ proportion matrix $U = [u_{p,(c,i)}]$ such that $f^{(c)}_{p,i} = \sum_{v(d,j) \in V(T_{(c,i)})} u_{p,(d,j)}$ for all character-state pairs (c, i) and all samples p.

Here, we show that the PCTI problem subject to the additional constraint that $J(U, U_1, U_2) = 0$ is equivalent to the CMPPD problem with $n = 2$ characters. Let (F_1, F_2, S_1, S_2) be a CMPPD problem instance the corresponding PCTI instance (T_1, U_1, T_2, U_2) has $T_1 = S_1$, $U_1 = F_1$, $T_2 = S_2$ and $U_2 = F_2$. Conversely, for a PCTI instance (T_1, U_1, T_2, U_2), the corresponding CMPPD instance (F_1, F_2, S_1, S_2) has $F_1 = U_1$, $F_2 = U_2$, $S_1 = T_1$ and $S_2 = T_2$. To see why this works observe that any clone tree T that is a refinement of clones T_1 and T_2 (as in Definition 3 in the main text) is also complete multi-state perfect phylogeny consistent with state trees T_1 and T_2, and vice versa. Thus, we can use SPRUCE [2] to enumerate all error-free solutions (if they exist) to any PCTI instance.

Previously, the CMPPD problem was shown to be NP-complete for $m = 2$ samples and state trees $\mathcal{S} = \{S_1, \ldots, S_n\}$ with two states each [2]. Here, we have shown that the decision version of the PCTI problem (i.e., deciding whether there exists a solution with $J(U, U_1, U_2) = 0$) is NP-complete (Theorem 5 in the main text). Based on the equivalence between the problems, an alternative hardness result follows for CMPPD.

Corollary 1. CMPPD is NP-complete even for $m = 1$ samples and $n = 2$ characters.

D MILP formulation for the PCTI problem

$$\min \sum_{p=1}^{m} \sum_{i=1}^{n_1} c^{(1)}_{p,i} + \sum_{p=1}^{m} \sum_{j=1}^{n_2} c^{(2)}_{p,j} \tag{2}$$

$$\text{s.t. } c^{(1)}_{p,i} \geq \sum_{j=1}^{n_2} u_{p,i,j} - u^{(1)}_{p,i} \qquad \forall p \in [m], i \in [n_1], \tag{3}$$

$$c^{(1)}_{p,i} \geq u^{(1)}_{p,i} - \sum_{j=1}^{n_2} u_{p,i,j} \qquad \forall p \in [m], i \in [n_1], \tag{4}$$

$$c_{p,j}^{(2)} \geq \sum_{i=1}^{n_1} u_{p,i,j} - u_{p,j}^{(2)} \qquad \forall p \in [m], j \in [n_2], \tag{5}$$

$$c_{p,j}^{(2)} \geq u_{p,j}^{(2)} - \sum_{i=1}^{n_1} u_{p,i,j} \qquad \forall p \in [m], j \in [n_2], \tag{6}$$

$$u_{p.i,j} \leq x_{i,j} \qquad \forall p \in [m], i \in [n_1], j \in [n_2], \tag{7}$$

$$\sum_{i=1}^{n_1}\sum_{j=1}^{n_2} u_{p,i,j} = 1 \qquad \forall p \in [m], \tag{8}$$

$$z_{(i,i'),j}^{(1)} \leq x_{i,j} \qquad \forall (i,i') \in E(T_1), j \in [n_2], \tag{9}$$

$$z_{(i,i'),j}^{(1)} \leq x_{i',j} \qquad \forall (i,i') \in E(T_1), j \in [n_2], \tag{10}$$

$$z_{(i,i'),j}^{(1)} \geq x_{i,j} + x_{i',j} - 1 \qquad \forall (i,i') \in E(T_1), j \in [n_2]. \tag{11}$$

$$z_{i,(j,j')}^{(2)} \leq x_{i,j} \qquad \forall i \in [n_1], (j,j') \in E(T_2), \tag{12}$$

$$z_{i,(j,j')}^{(2)} \leq x_{i,j'} \qquad \forall i \in [n_1], (j,j') \in E(T_2), \tag{13}$$

$$z_{i,(j,j')}^{(2)} \geq x_{i,j} + x_{i,j'} - 1 \qquad \forall i \in [n_1], (j,j') \in E(T_2), \tag{14}$$

$$\sum_{j=1}^{n_2} z_{(i,i'),j}^{(1)} = 1 \qquad \forall (i,i') \in E(T_1), \tag{15}$$

$$\sum_{i=1}^{n_1} z_{i,(j,j')}^{(2)} = 1 \qquad \forall (j,j') \in E(T_2), \tag{16}$$

$$x_{i,j} \in \{0,1\}, \qquad \forall i \in [n_1], j \in [n_2], \tag{17}$$

$$u_{p,i}^{(1)} \in [0,1], \qquad \forall p \in [m], i \in [n_1], \tag{18}$$

$$u_{p,j}^{(2)} \in [0,1], \qquad \forall p \in [m], j \in [n_2], \tag{19}$$

$$c_{p,i}^{(1)} \in [0,1], \qquad \forall p \in [m], i \in [n_1], \tag{20}$$

$$c_{p,j}^{(2)} \in [0,1], \qquad \forall p \in [m], j \in [n_2], \tag{21}$$

$$z_{(i,i'),j}^{(1)} \geq 0 \qquad \forall (i,i') \in E(T_1), j \in [n_2], \tag{22}$$

$$z_{i,(j,j')}^{(2)} \geq 0 \qquad \forall i \in [n_1], (j,j') \in E(T_2). \tag{23}$$

E Simulation Details

We perturb the proportion matrices U_1 and U_2 by introducing noise following a user-defined level $h \in [0,1]$. For each sample $p \in [m]$, let $\mathbf{u}_p^{(1)} = [u_{p,i}^{(1)}]$ for $i \in [n_1]$ and $\mathbf{u}_p^{(2)} = [u_{p,j}^{(2)}]$ for $j \in [n_2]$. The perturbed proportions $\bar{\mathbf{u}}_p^{(1)}$ and $\bar{\mathbf{u}}_p^{(2)}$ are drawn from the following distributions

$$\bar{\mathbf{u}}_p^{(1)} \sim (1-h)\mathbf{u}_p^{(1)} + h\text{Dir}(\mathbf{1}_{n_1}), \quad \forall p \in [m],$$

$$\bar{\mathbf{u}}_p^{(2)} \sim (1-h)\mathbf{u}_p^{(2)} + h\text{Dir}(\mathbf{1}_{n_2}), \quad \forall p \in [m].$$

The resulting proportion matrices are $\bar{U}_1 = [\bar{u}_{p,i}^{(1)}]$ for $p \in [m], i \in [n_1]$ and $\bar{U}_2 = [\bar{u}_{p,j}^{(2)}]$ for $p \in [m], j \in [n_2]$. Note that when noise level $h = 0$, we have $\bar{U}_1 = U_1$ and $\bar{U}_2 = U_2$. Also, for any $h \in [0,1]$, the matrices $\bar{U}_1$ and $\bar{U}_2$ satisfy the conditions laid out in the definition of proportion matrices (Definition 2 in the main text).

F Computation of SNV Clone Proportions

Each edge of the SNV clone tree T_1 reported by Gundem *et al.* [5] represents a set of mutations, also known as mutation clusters. As such, for a SNV clone tree T_1 with n_1 vertices, there are $n_1 - 1$ mutation clusters. The authors have provided the cancer cell fraction (CCF) for each of the mutation clusters in each sample of the ten patients. They used pigeonhole principle (PPH) to construct the SNV clone tree manually. For a given patient, let $F \in [0,1]^{m \times (n_1 - 1)}$ be the CCF matrix such that $F = [f_{p,k}]$ and $f_{p,k}$ is the CCF of mutation cluster $k \in [n_1 - 1]$ in sample $p \in [m]$. The SNV clone tree T_1, excluding the root vertex which represent the normal cell, is used to construct a perfect phylogeny matrix B [6]. We use the perfect phylogeny matrix B and the CCF matrix F to get the proportion U' of SNV clones, excluding the normal clone, in each sample of the ten patients by solving the following linear program

$$\begin{aligned}
&\min |F - BU'|_1, \\
&\text{s.t. } 0 \le u_{p,i} \le 1, \quad \forall p \in [m], i \in [n_1 - 1], \\
&\qquad \sum_{i=1}^{n_1 - 1} u_{p,i} = 1, \quad \forall p \in [m],
\end{aligned}$$

where $|\cdot|_1$ is the entry-wise L_1 norm. Finally, we correct the proportion matrix U' for the purity of the tumor samples (also known as tumor cellularity), which is the proportion of cancer cells in the tumor. We use the proportion of normal cells in each sample, inferred by HATCHet [7], to compute the purity of the tumor samples. Let $\boldsymbol{\gamma} \in [0,1]^{m \times 1}$ be a vector such that $\gamma_{p,1}$ is the purity of sample $p \in [m]$ inferred using HATCHet. The proportion matrix $U \in [0,1]^{m \times n_1}$ of the SNV clones is given by

$$U = \begin{bmatrix} \text{Diag}(\boldsymbol{\gamma})U' & \mathbf{1}_m - \boldsymbol{\gamma} \end{bmatrix}$$

where $\mathbf{1}_m$ is a $m \times 1$ vector with all entries equal to 1 and $\text{Diag}(\gamma)$ is a $m \times m$ diagonal matrix with the diagonal elements given by the entries of the vector $\boldsymbol{\gamma}$. It is easy to see that the proportion matrix U satisfies the conditions for being a proportion matrix (see Definition 1 in the main text).

G Supplementary Figures and Tables

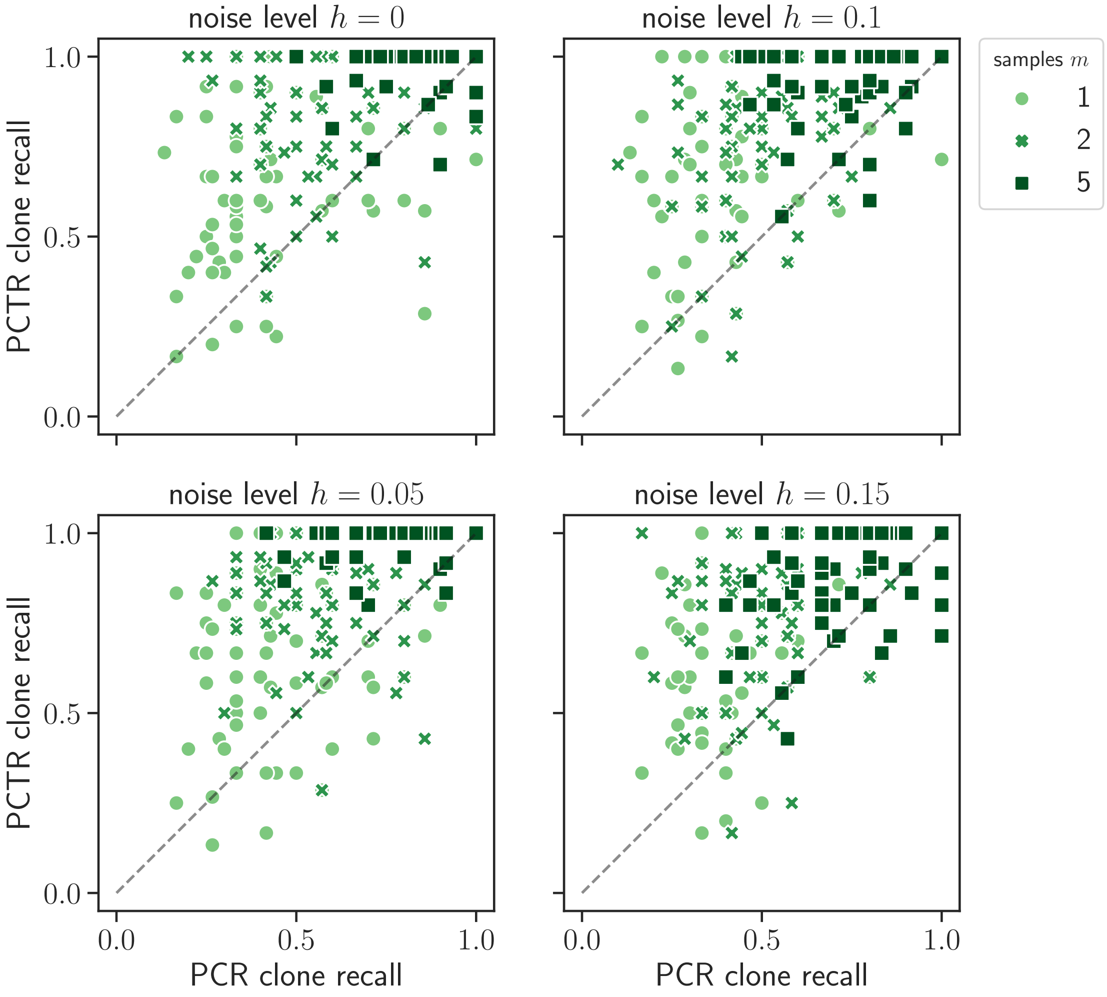

Figure S1: Clone recall for the two modes of PACTION on the simulated instances. We show the clone recall of PACTION with the PCI and the PCTI mode on the simulated instances for varying noise levels h and number m of samples. For majority of simulated instances, PACTION in the PCTI mode has a higher recall compared to the PCI mode.

number of samples m	PCI runtime (s)	PCTI runtime (s)
1	0.84820	0.74365
2	0.6949	0.7379
5	0.81985	0.84460

Table S1: Median running time of PACTION in PCI and PCTI modes for simulation instances with varying number of samples m.

patient	number m of samples	number n_1 of SNV clones	number n_2 of CNA clones
A10	4	10	8
A12	3	5	8
A17	5	11	6
A21	8	15	6
A22	10	16	4
A24	4	10	4
A29	2	6	4
A31	5	11	6
A32	5	13	6
A34	3	14	6

Table S2: Statistics of the metastatic prostate cancer data [5]. Number m of samples, number n_1 of SNV clones and number n_2 of CNA clones for the 10 patients from Gundem *et al.* [5]. The CNA clones were identified using HATCHet [7].

References

[1] Ravindra K Ahuja, Thomas L Magnanti, James B Orlin, and K Weihe. Network flows: theory, algorithms and applications. *ZOR-methods and models of operations research*, 41(3):252–254, 1995.

[2] Mohammed El-Kebir, Gryte Satas, Layla Oesper, and Benjamin J. Raphael. Inferring the Mutational History of a Tumor Using Multi-state Perfect Phylogeny Mixtures. *Cell Systems*, 3(1):43–53, July 2016.

[3] D. Gusfield. *ReCombinatorics: The Algorithmics of Ancestral Recombination Graphs and Explicit Phylogenetic Networks*. MIT Press, 2014.

[4] D Fernández-Baca. The perfect phylogeny problem. In D Z Zu and X Cheng, editors, *Steiner Trees in Industries*. Kluwer Acedemic Publishers, 2000.

[5] Gunes Gundem, Peter Van Loo, Barbara Kremeyer, Ludmil B Alexandrov, Jose MC Tubio, Elli Papaemmanuil, Daniel S Brewer, Heini ML Kallio, Gunilla Högnäs, Matti Annala, et al. The evolutionary history of lethal metastatic prostate cancer. *Nature*, 520(7547):353–357, 2015.

[6] Dikshant Pradhan and Mohammed El-Kebir. On the non-uniqueness of solutions to the perfect phylogeny mixture problem. In *RECOMB International conference on Comparative Genomics*, pages 277–293. Springer, 2018.

[7] Simone Zaccaria and Benjamin J Raphael. Accurate quantification of copy-number aberrations and whole-genome duplications in multi-sample tumor sequencing data. *Nature communications*, 11(1):1–13, 2020.
